# Supplementary material for: A comprehensive descriptive analysis of hip and knee radiographic osteoarthritis in the UK Biobank in relation to joint pain, joint site interrelationships, obesity, race and deprivation: Findings from 59,475 individuals
Source: medRxiv. 2026 Mar 17:2026.03.15.26348416. Preprint. [Version 1] doi: 10.64898/2026.03.15.26348416 (PMC13015658; doi:10.64898/2026.03.15.26348416)
Supplement: Supplement 1 [file media-1.docx]

Supplementary Table 1 - Logistic regression results showing the associations between increasing grades of radiographic osteoarthritis and self-reported pain

|  |  | **Odds of hip Pain** | |  | **Odds of knee pain** | | | |
| --- | --- | --- | --- | --- | --- | --- | --- | --- |
|  | **Unadjusted** | | **Fully Adjusted** | | **Unadjusted** | | **Fully Adjusted** | |
|  | **OR [95% CI]** | **P** | **OR [95% CI]** | **P** | **OR [95% CI]** | **P** | **OR [95% CI]** | **P** |
| **Right rHOA 1** | 0.99 [0.90 - 1.09] | 0.79 | 1.08 [0.98 - 1.19] | 0.14 | 1.12 [1.04 - 1.20] | 1.57 × 10^-3^ | 1.16 [1.08 - 1.25] | 4.37 × 10^-5^ |
| **Right rHOA 2** | 1.72 [1.54 - 1.92] | 2.11 × 10^-22^ | 1.89 [1.69 - 2.11] | 9.32 × 10^-29^ | 1.06 [0.96 - 1.17] | 0.22 | 1.04 [0.94 - 1.15] | 0.44 |
| **Right rHOA 3** | 4.23 [3.55 - 5.03] | 2.50 × 10^-59^ | 4.55 [3.81 - 5.44] | 1.80 × 10^-62^ | 1.29 [1.06 - 1.57] | 0.01 | 1.25 [1.02 - 1.52] | 0.03 |
| **Right rHOA 4** | 13.44 [10.01 - 18.04] | 5.60 × 10^-67^ | 15.87 [11.75 - 21.43] | 1.40 × 10^-72^ | 1.57 [1.10 - 2.25] | 0.01 | 1.58 [1.10 - 2.27] | 0.01 |
|  |  |  |  |  |  |  |  |  |
| **Left rHOA 1** | 1.00 [0.91 - 1.10] | 0.98 | 1.09 [1.00 - 1.20] | 0.06 | 1.13 [1.06 - 1.21] | 4.32 × 10^-4^ | 1.16 [1.09 - 1.25] | 1.92 × 10^-5^ |
| **Left rHOA 2** | 1.41 [1.26 - 1.57] | 6.26 × 10^-10^ | 1.58 [1.41 - 1.76] | 1.10 × 10^-15^ | 1.28 [1.17 - 1.40] | 2.45 × 10^-8^ | 1.25 [1.14 - 1.37] | 9.63 × 10^-7^ |
| **Left rHOA 3** | 3.14 [2.64 - 3.73] | 1.02 × 10^-38^ | 3.63 [3.04 - 4.33] | 1.40 × 10^-46^ | 1.26 [1.05 - 1.51] | 0.01 | 1.25 [1.03 - 1.50] | 0.02 |
| **Left rHOA 4** | 9.40 [7.17 - 12.31] | 1.80 × 10^-59^ | 11.37 [8.63 - 14.99] | 7.60 × 10^-67^ | 1.94 [1.42 - 2.64] | 3.10 × 10^-5^ | 2.02 [1.47 - 2.76] | 1.32 × 10^-5^ |
|  |  |  |  |  |  |  |  |  |
| **Right rKOA 1** | 1.17 [1.07 - 1.27] | 3.47 × 10^-4^ | 0.96 [0.88 - 1.04] | 0.31 | 2.09 [1.96 - 2.22] | 2.00 × 10^-121^ | 2.04 [1.91 - 2.18] | 2.00 × 10^-105^ |
| **Right rKOA 2** | 1.27 [1.13 - 1.44] | 1.01 × 10^-4^ | 0.98 [0.86 - 1.11] | 0.73 | 4.17 [3.85 - 4.50] | 2.00 × 10^-279^ | 3.82 [3.52 - 4.15] | 4.00 × 10^-227^ |
| **Right rKOA 3** | 1.30 [0.95 - 1.76] | 0.10 | 0.93 [0.68 - 1.27] | 0.65 | 9.33 [7.74 - 11.26] | 5.00 × 10^-121^ | 8.04 [6.64 - 9.74] | 6.00 × 10^-101^ |
| **Right rKOA 4** | 0.98 [0.57 - 1.69] | 0.95 | 0.60 [0.34 - 1.04] | 0.07 | 12.10 [8.93 - 16.40] | 2.70 × 10^-58^ | 8.99 [6.58 - 12.28] | 3.10 × 10^-43^ |
|  |  |  |  |  |  |  |  |  |
| **Left rKOA 1** | 1.14 [1.05 - 1.24] | 1.19 × 10^-3^ | 0.94 [0.87 - 1.03] | 0.17 | 1.98 [1.87 - 2.11] | 6.00 × 10^-111^ | 1.93 [1.81 - 2.06] | 9.20 × 10^-95^ |
| **Left rKOA 2** | 1.22 [1.09 - 1.38] | 6.99 × 10^-4^ | 0.93 [0.82 - 1.04] | 0.21 | 4.02 [3.73 - 4.34] | 3.00 × 10^-289^ | 3.66 [3.39 - 3.96] | 5.00 × 10^-231^ |
| **Left rKOA 3** | 1.16 [0.86 - 1.58] | 0.33 | 0.80 [0.59 - 1.10] | 0.17 | 9.48 [7.93 - 11.33] | 2.00 × 10^-134^ | 7.92 [6.59 - 9.51] | 1.00 × 10^-108^ |
| **Left rKOA 4** | 1.32 [0.84 - 2.08] | 0.23 | 0.88 [0.56 - 1.40] | 0.60 | 17.76 [13.14 - 24.01] | 4.10 × 10^-78^ | 14.60 [10.74 - 19.83] | 7.80 × 10^-66^ |

*Adjusted for age, sex, race, height, weight and deprivation*

*rHOA – radiographic hip osteoarthritis, rKOA – radiographic knee osteoarthritis, OR – odds ratio, P – p-value*

Supplementary Table 2 - Logistic regression results for the odds of osteoarthritis based on presence at each joint in combined and sex-stratified analyses

|  | **COMBINED** | | | | **MALE** | | | | **FEMALE** | | | |
| --- | --- | --- | --- | --- | --- | --- | --- | --- | --- | --- | --- | --- |
|  | **Unadjusted** | | **Fully Adjusted** | | **Unadjusted** | | **Fully Adjusted** | | **Unadjusted** | | **Fully Adjusted** | |
|  | **OR [95% CI]** | **P** | **OR [95% CI]** | **P** | **OR [95% CI]** | **P** | **OR [95% CI]** | **P** | **OR [95% CI]** | **P** | **OR [95% CI]** | **P** |
| Right rHOA |  |  |  |  |  |  |  |  |  |  |  |  |
| **Odds of left hip rOA** | 6.66 [6.18 - 7.17] | <0.01 × 10^-324^ | 5.64 [5.23 - 6.09] | <0.01 × 10^-324^ | 5.57 [5.09 - 6.10] | 1.00 × 10^-301^ | 5.37 [4.90 - 5.88] | 4.00 × 10^-285^ | 7.17 [6.28 - 8.20] | 5.00 × 10^-185^ | 6.11 [5.33 - 7.00] | 8.00 × 10^-150^ |
| **Odds of right knee rOA** | 1.31 [1.17 - 1.48] | 7.11 × 10^-6^ | 1.25 [1.10 - 1.41] | 4.15 × 10^-4^ | 1.29 [1.08 - 1.55] | 5.99 × 10^-3^ | 1.16 [0.97 - 1.39] | 0.11 | 1.65 [1.41 - 1.94] | 5.70 × 10^-10^ | 1.32 [1.12 - 1.56] | 8.27 × 10^-4^ |
| **Odds of Left knee rOA** | 1.41 [1.26 - 1.57] | 1.04 × 10^-9^ | 1.36 [1.22 - 1.53] | 7.96 × 10^-8^ | 1.38 [1.16 - 1.63] | 1.98 × 10^-4^ | 1.25 [1.06 - 1.48] | 9.88 × 10^-3^ | 1.79 [1.55 - 2.08] | 1.02 × 10^-14^ | 1.47 [1.26 - 1.71] | 8.78 × 10^-7^ |
| Left rHOA |  |  |  |  |  |  |  |  |  |  |  |  |
| **Odds of right hip rOA** | 6.66 [6.18 - 7.17] | <0.01 × 10^-324^ | 5.64 [5.22 - 6.08] | <0.01 × 10^-324^ | 5.57 [5.09 - 6.10] | 1.00 × 10^-301^ | 5.37 [4.90 - 5.88] | 3.00 × 10^-285^ | 7.17 [6.28 - 8.20] | 5.00 × 10^-185^ | 6.12 [5.34 - 7.01] | 8.00 × 10^-150^ |
| **Odds of right knee rOA** | 1.26 [1.13 - 1.41] | 5.14 × 10^-5^ | 1.31 [1.16 - 1.47] | 5.50 × 10^-6^ | 1.40 [1.20 - 1.65] | 3.21 × 10^-5^ | 1.29 [1.10 - 1.52] | 1.92 × 10^-3^ | 1.57 [1.34 - 1.85] | 3.17 × 10^-8^ | 1.32 [1.12 - 1.56] | 9.25 × 10^-4^ |
| **Odds of left knee rOA** | 1.33 [1.20 - 1.47] | 9.91 × 10^-8^ | 1.40 [1.25 - 1.56] | 1.28 × 10^-9^ | 1.41 [1.21 - 1.64] | 9.43 × 10^-6^ | 1.30 [1.12 - 1.52] | 6.58 × 10^-4^ | 1.75 [1.51 - 2.03] | 1.07 × 10^-13^ | 1.50 [1.29 - 1.74] | 1.84 × 10^-7^ |
| Right rKOA |  |  |  |  |  |  |  |  |  |  |  |  |
| **Odds of right hip rOA** | 1.31 [1.17 - 1.48] | 7.11 × 10^-6^ | 1.27 [1.13 - 1.44] | 1.28 × 10^-9^ | 1.29 [1.08 - 1.55] | 5.99 × 10^-3^ | 1.16 [0.97 - 1.40] | 0.11 | 1.65 [1.41 - 1.94] | 5.70 × 10^-10^ | 1.31 [1.12 - 1.55] | 1.07 × 10^-3^ |
| **Odds of left hip rOA** | 1.26 [1.13 - 1.41] | 5.14 × 10^-5^ | 1.32 [1.18 - 1.48] | 1.91 × 10^-6^ | 1.40 [1.20 - 1.65] | 3.21 × 10^-5^ | 1.29 [1.10 - 1.52] | 2.02 × 10^-3^ | 1.57 [1.34 - 1.85] | 3.17 × 10^-8^ | 1.31 [1.11 - 1.55] | 1.29 × 10^-3^ |
| **Odds of left knee rOA** | 32.80 [30.34 - 35.45] | <0.01 × 10^-324^ | 26.07 [24.07 - 28.24] | <0.01 × 10^-324^ | 39.32 [34.37 - 44.97] | <0.01 × 10^-324^ | 33.50 [29.21 - 38.43] | <0.01 × 10^-324^ | 27.81 [25.28 - 30.61] | <0.01 × 10^-324^ | 22.91 [20.77 - 25.27] | <0.01 × 10^-324^ |
| Left rKOA |  |  |  |  |  |  |  |  |  |  |  |  |
| **Odds of right hip rOA** | 1.41 [1.26 - 1.57] | 1.04 × 10^-9^ | 1.39 [1.24 - 1.55] | 1.32 × 10^-8^ | 1.38 [1.16 - 1.63] | 1.98 × 10^-4^ | 1.25 [1.06 - 1.49] | 9.16 × 10^-3^ | 1.79 [1.55 - 2.08] | 1.02 × 10^-14^ | 1.46 [1.25 - 1.70] | 1.17 × 10^-6^ |
| **Odds of left hip rOA** | 1.33 [1.20 - 1.47] | 9.91 × 10^-8^ | 1.41 [1.27 - 1.57] | 3.35 × 10^-10^ | 1.41 [1.21 - 1.64] | 9.43 × 10^-6^ | 1.30 [1.12 - 1.52] | 7.16 × 10^-4^ | 1.75 [1.51 - 2.03] | 1.07 × 10^-13^ | 1.49 [1.28 - 1.73] | 2.99 × 10^-7^ |
| **Odds of right knee rOA** | 32.80 [30.34 - 35.45] | <0.01 × 10^-324^ | 26.09 [24.09 - 28.26] | <0.01 × 10^-324^ | 39.32 [34.37 - 44.97] | <0.01 × 10^-324^ | 33.55 [29.25 - 38.49] | <0.01 × 10^-324^ | 27.81 [25.28 - 30.61] | <0.01 × 10^-324^ | 22.93 [20.79 - 25.30] | <0.01 × 10^-324^ |

*Adjusted for age, sex, race, height, weight and deprivation*

*rHOA – radiographic hip osteoarthritis, rKOA – radiographic knee osteoarthritis, OR – odds ratio, CI – confidence interval, P – p-value*

Supplementary Table 3 - Prevalence of radiographic osteoarthritis among the different quintiles of deprivation (TDI) and racial background

|  | **Right rHOA** | **Left rHOA** | **Right rKOA** | **Left rKOA** |
| --- | --- | --- | --- | --- |
|  | **Frequency [%]** | **Frequency [%]** | **Frequency [%]** | **Frequency [%]** |
| TDI quintiles |  |  |  |  |
| **1 (least deprived)** | 1803 [7.21] | 2,067 [8.27] | 1553 [6.21] | 1780 [7.12] |
| **2** | 969 [6.52] | 1,195 [8.04] | 948 [6.38] | 1068 [7.19] |
| **3** | 695 [6.88] | 765 [7.57] | 648 [6.41] | 686 [6.79] |
| **4** | 436 [6.62] | 559 [8.49] | 432 [6.56] | 485 [7.37] |
| **5 (most deprived)** | 195 [6.66] | 225 [8.71] | 169 [5.78] | 201 [6.87] |
| Race |  |  |  |  |
| **White** | 3,766 [6.87] | 4,430 [8.08] | 3,423 [6.24] | 3880 [7.08] |
| **Asian** | 43 [6.61] | 54 [8.29] | 44 [6.76] | 47 [7.22] |
| **Black** | 14 [3.54] | 35 [8.86] | 35 [8.86] | 40 [10.13] |
| **Mixed** | 11 [3.70] | 17 [5.72] | 22 [7.41] | 25 [8.42] |
| **Chinese** | 5 [3.25] | 5 [3.25] | 9 [5.84] | 12 [7.79] |
| **Unknown** | 259 [8.24] | 300 [9.55] | 217 [6.91] | 216 [6.87] |

*TDI – Townsend Deprivation Index, rHOA – radiographic hip osteoarthritis, rKOA – radiographic knee osteoarthritis*

Supplementary Table 4 - Binary logistic regression for the associations between radiographic osteoarthritis and quintiles of deprivation (TDI)

|  | **Unadjusted** | | **Fully Adjusted** | |
| --- | --- | --- | --- | --- |
|  | **OR [95% CI]** | **P** | **OR [95% CI]** | **P** |
| Right rHOA |  |  |  |  |
| **TDI quintile 2** | 0.90 [0.83 - 0.97] | 8.60 × 10^-3^ | 0.92 [0.85 - 1.00] | 0.05 |
| **TDI quintile 3** | 0.95 [0.87 - 1.04] | 0.27 | 1.01 [0.92 - 1.10] | 0.87 |
| **TDI quintile 4** | 0.91 [0.82 - 1.02] | 0.10 | 0.98 [0.88 - 1.10] | 0.75 |
| **TDI quintile 5** | 0.92 [0.79 - 1.07] | 0.28 | 1.02 [0.87 - 1.19] | 0.84 |
| Left rHOA |  |  |  |  |
| **TDI quintile 2** | 0.97 [0.90 - 1.04] | 0.42 | 0.99 [0.92 - 1.07] | 0.87 |
| **TDI quintile 3** | 0.91 [0.83 - 0.99] | 0.03 | 0.95 [0.87 - 1.04] | 0.26 |
| **TDI quintile 4** | 1.03 [0.93 - 1.13] | 0.56 | 1.09 [0.99 - 1.21] | 0.08 |
| **TDI quintile 5** | 1.06 [0.92 - 1.21] | 0.41 | 1.14 [0.99 - 1.31] | 0.07 |
| Right rKOA |  |  |  |  |
| **TDI quintile 2** | 1.03 [0.95 - 1.12] | 0.51 | 1.01 [0.93 - 1.10] | 0.74 |
| **TDI quintile 3** | 1.03 [0.94 - 1.14] | 0.48 | 1.03 [0.93 - 1.13] | 0.55 |
| **TDI quintile 4** | 1.06 [0.95 - 1.18] | 0.30 | 1.03 [0.92 - 1.16] | 0.56 |
| **TDI quintile 5** | 0.93 [0.79 - 1.09] | 0.35 | 0.91 [0.77 - 1.07] | 0.25 |
| Left rKOA |  |  |  |  |
| **TDI quintile 2** | 1.01 [0.93 - 1.09] | 0.81 | 0.99 [0.91 - 1.07] | 0.83 |
| **TDI quintile 3** | 0.95 [0.87 - 1.04] | 0.27 | 0.94 [0.85 - 1.03] | 0.18 |
| **TDI quintile 4** | 1.04 [0.93 - 1.15] | 0.49 | 1.01 [0.90 - 1.12] | 0.92 |
| **TDI quintile 5** | 0.96 [0.83 - 1.12] | 0.62 | 0.94 [0.80 - 1.09] | 0.41 |

*Adjusted for age, sex, race, height and weight*

*TDI – Townsend Deprivation Index, OR – odds ratio, CI – confidence interval, rHOA – radiographic hip osteoarthritis, rKOA – radiographic knee osteoarthritis, P – p-value*

Supplementary Table 5 – Binary logistic regression for the associations between radiographic osteoarthritis and racial groups

|  | **Unadjusted** | | **Fully Adjusted** | |
| --- | --- | --- | --- | --- |
|  | **OR [95% CI]** | **P** | **OR [95% CI]** | **P** |
| Right rHOA |  |  |  |  |
| **Asian** | 0.96 [0.70 - 1.31] | 0.79 | 1.19 [0.87 - 1.63] | 0.28 |
| **Black** | 0.50 [0.29 - 0.85] | 0.01 | 0.64 [0.37 - 1.10] | 0.11 |
| **Mixed** | 0.52 [0.29 - 0.95] | 0.03 | 0.71 [0.39 - 1.31] | 0.28 |
| **Chinese** | 0.46 [0.19 - 1.11] | 0.08 | 0.73 [0.30 - 1.79] | 0.49 |
| **Unknown** | 1.22 [1.07 - 1.39] | 3.00 × 10^-3^ | 1.15 [1.01 - 1.31] | 0.04 |
| Left rHOA |  |  |  |  |
| **Asian** | 1.03 [0.78 - 1.36] | 0.84 | 1.17 [0.88 - 1.56] | 0.28 |
| **Black** | 1.11 [0.78 - 1.57] | 0.57 | 1.32 [0.92 - 1.88] | 0.13 |
| **Mixed** | 0.69 [0.42 - 1.13] | 0.14 | 0.91 [0.56 - 1.50] | 0.72 |
| **Chinese** | 0.38 [0.16 - 0.93] | 0.03 | 0.58 [0.24 - 1.43] | 0.24 |
| **Unknown** | 1.20 [1.06 - 1.36] | 3.50 × 10^-3^ | 1.18 [1.04 - 1.34] | 0.01 |
| Right rKOA |  |  |  |  |
| **Asian** | 1.09 [0.80 - 1.48] | 0.59 | 1.65 [1.20 - 2.26] | 2.00 × 10^-3^ |
| **Black** | 1.46 [1.03 - 2.07] | 0.03 | 1.43 [0.99 - 2.06] | 0.05 |
| **Mixed** | 1.20 [0.78 - 1.86] | 0.41 | 1.29 [0.82 - 2.02] | 0.27 |
| **Chinese** | 0.93 [0.48 - 1.83] | 0.84 | 1.58 [0.80 - 3.14] | 0.19 |
| **Unknown** | 1.11 [0.97 - 1.28] | 0.14 | 0.90 [0.78 - 1.04] | 0.16 |
| Left rKOA |  |  |  |  |
| **Asian** | 1.02 [0.76 - 1.38] | 0.89 | 1.55 [1.14 - 2.10] | 5.20 × 10^-3^ |
| **Black** | 1.48 [1.07 - 2.06] | 0.02 | 1.43 [1.01 - 2.01] | 0.04 |
| **Mixed** | 1.21 [0.80 - 1.82] | 0.37 | 1.27 [0.83 - 1.95] | 0.26 |
| **Chinese** | 1.11 [0.62 - 2.00] | 0.73 | 1.89 [1.04 - 3.44] | 0.04 |
| **Unknown** | 0.97 [0.84 - 1.12] | 0.67 | 0.80 [0.69 - 0.92] | 2.30 × 10^-3^ |

*Adjusted for age, sex, height, weight and deprivation*

*White race as the comparator group*

*rHOA – radiographic hip osteoarthritis, rKOA – radiographic knee osteoarthritis, OR – odds ratio, CI – confidence interval, P – p-value*


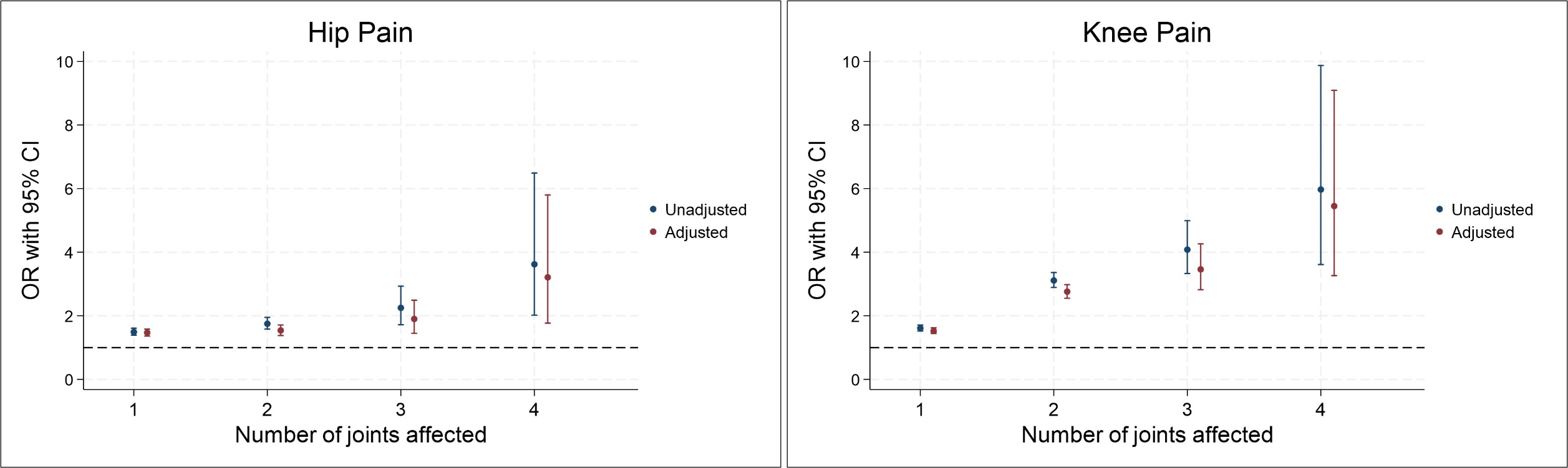


Supplementary Figure 1 - Logistic regression results for the associations between number of joints affected and self-reported hip or knee pain.

*Adjusted for age, sex, race, height, weight and deprivation*


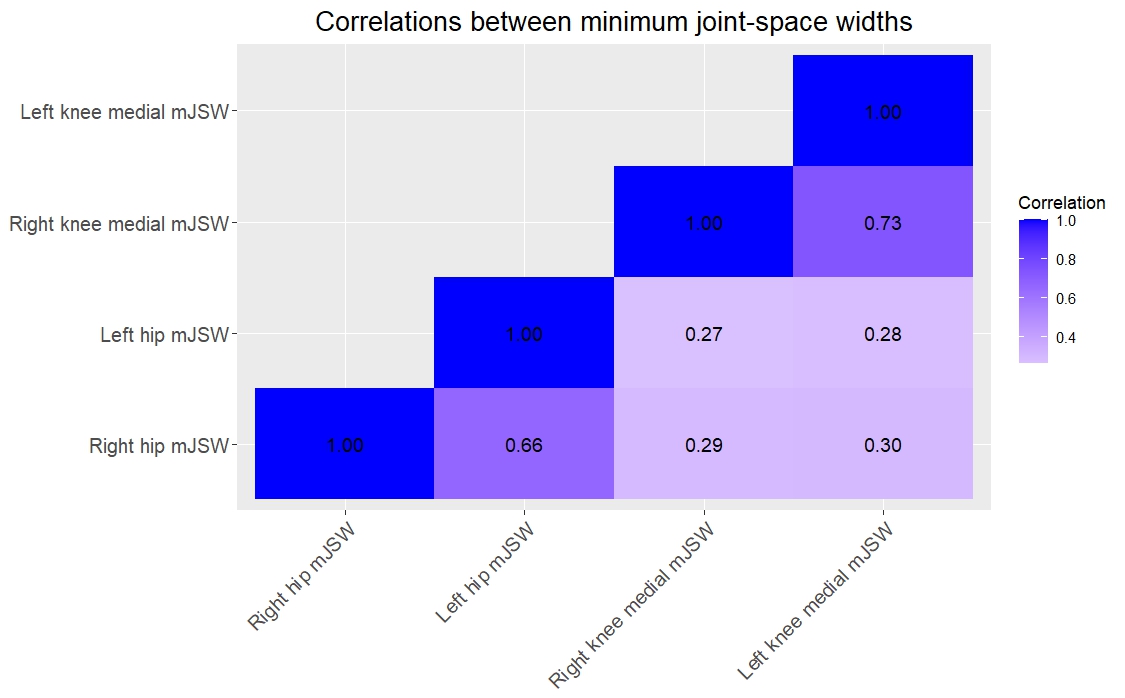


Supplementary Figure 2 - Correlations matrix to visually display the relationships between the minimum joint-space widths at each of the four anatomical joints

*mJSW- minimum joint-space width*
